# Terminally sialylated and fucosylated complex N-glycans are involved in the malignant behavior of high-grade glioma

**DOI:** 10.18632/oncotarget.27850

**Published:** 2020-12-29

**Authors:** Hector A. Cuello, Gretel M. Ferreira, Cynthia A. Gulino, Alejandro Gomez Toledo, Valeria I. Segatori, Mariano R. Gabri

**Affiliations:** ^1^Center for Molecular and Translational Oncology, Quilmes National University, Bernal, Buenos Aires Province, Argentina; ^2^Infection Medicine (BMC), Faculty of Medicine, Department of Clinical Sciences, Lund University, Lund, Sweden

**Keywords:** glioma, glioblastoma, Lewis glycans, histone acetylation, N-glycans

## Abstract

Gliomas are the most common intracranial primary tumors, for which very few therapeutic options are available. The most malignant subtype is the glioblastoma, a disease associated with a 5-year survival rate lower than 5%. Given that research in glycobiology continues highlighting the role of glycans in tumor cell biology, it offers an interesting niche for the search of new therapeutic targets. In this study, we characterized aberrant glycosylation and its impact on cell biology over a broad panel of high- and low-grade glioma cell lines. Results show high expression of terminal Lewis glycans, mainly SLe^x^, and overexpression of sialyl- and fucosyltransferases involved in their biosynthesis in high-grade glioma cell lines. Moreover, we report an association of complex multi-antennary N-glycans presenting β1,6-GlcNAc branches with the high-grade glioma cells, which also overexpressed the gene responsible for these assemblies, MGAT5. In addition, downmodulation of N-glycosylation by treatment with the inhibitors Tunicamycin/Swainsonine or MGAT5 silencing decreased SLe^x^ expression, adhesion and migration in high-grade glioma cells. In contrast, no significant changes in these cell capacities were observed in low-grade glioma after treatment with the N-glycosylation inhibitors. Furthermore, inhibition of histone deacetylases by Trichostatin A provoked an increase in the expression of SLe^x^ and its biosynthetic related glycosyltransferases in low-grade glioma cells. Our results describe that aggressive glioma cells show high expression of Lewis glycans anchored to complex multi-antennary N-glycans. This glycophenotype plays a key role in malignant cell behavior and is regulated by histone acetylation dependent mechanisms.

## INTRODUCTION

Gliomas are located in the central nervous system and derive from glial cell precursors. They are classified by the World Health Organization (WHO) into four groups according to aggressiveness parameters, the grade IV or glioblastoma (GBM) being the most malignant subtype for which no cure is available [1]. 46.6% of all adult gliomas are GBMs, with an average incidence of 2.47 cases per 100,000 in the USA. Although novel clinical options have been approved in the last years, about 50% of the patients do not survive the first year after diagnosis and only a meager 5% reach 5-year survival rates [2]. In this scenario, research in glycobiology emerges as a promising niche to find novel targets for clinical application in glioma.

Glycans, both alone and in conjugation with other macromolecules, are essential for the existence of all multicellular cells. They play a key role in structural/modulatory functions and also in cell-cell and cell-microenvironment interactions [3]. Although significant progress has been made in understanding the role of glycans in several cancer indications, the impact of glycosylation and the potential of glycans as therapeutic targets merit further study [4].

Except for hyaluronic acid and glycogen, glycans are always attached to protein or lipid to form glycoconjugates through a process called glycosylation [5]. The major types of protein glycosylation include N-linked (Asn-linked) and O-linked (Ser/Thr-linked) glycans. The repertoire of glycans expressed in a single cell is known as the glycome, determined by multiple components of the glycosylation machinery, mainly by the array of glycosyltransferases and glycosidases expressed. A glycome is in fact the result of a non-templated process regulated at different levels, from gene expression to modulations at multiple steps in the endoplasmic reticulum (ER) and Golgi apparatus. In this regard, epigenetic regulation of genes involved in protein glycosylation is one of the mechanisms in which research is still scarce [6]. Among them, histone acetylation regulates gene transcription in a process that involves histone acetyltransferases (HATs) and histone deacetylases (HDACs). In particular, HDACs are related to repression of genes that encode proteins involved in tumorigenesis, specifically in control of cell growth, differentiation and apoptosis [7]. The association of HDACs with glycosylation patterns has been reported for cancer indications such as neuroblastoma and adrenocortical carcinoma [8, 9].

Aberrant glycosylation is a phenomenon involving changes in the glycosylation profile of normal cell progenitors and is concomitant with the multistep process of malignant transformation [10]. An altered glycosylation profile has been associated with cellular features that promote tumor progression, such as adhesion to the extracellular matrix, migration, invasion, inhibition of apoptosis, host immunoregulation and resistance to chemotherapy [11]. Cancer-associated structures, mainly reported in carcinomas, include truncated O-GalNAc glycans, Lewis glycans such as SLe^x^ and alterations in O- and N-glycans branching [12].

In the same line, the glycosyltransferases responsible for this aberrant pattern of glycosylation have been associated with cancer. Particularly, Lewis glycans are a family of related structures found at the terminal end of the glycan chains on cell surface glycoconjugates whose biosynthesis involves several glycosyltransferases, namely β1,3/4-galactosyltransferases (B3/4GalT), α1,2-fucosyltransferases (Fut1/2), α1,3-fucosyltransferases (FuT3-7 and 9-11) and α2,3-sialyltransferases (ST3Gal3/4/6) [13–15]. The presence of these glycans in tumors is mainly associated with overexpression or *de novo* synthesis, principally due to alterations in fucosylation and sialylation processes [12]. Regarding branching, the core 2 β1,6-N-acetylglucosaminyltransferase 1 (C2GnT1) encoded by C2GNT1 gene is the main enzyme involved in the synthesis of core 2 in O-GalNAc glycans, and the N-acetylglucosaminyltransferase V (GnT-V) encoded by MGAT5 gene is responsible for β1,6-GlcNAc branching in N-glycans [16, 17]. Both types of branching are potential scaffolds for such terminal assemblies as the Lewis glycans. Moreover, high expression of both core 2 O-GalNAc glycans and N-glycans bearing β1,6-GlcNAc branching has been associated with aggressiveness and tumor progression in several types of cancer [8, 18–26].

Even though gliomas are of particular interest to many research groups and the pharmaceutical industry, little is known about their glycobiology. Data have shown high expression of glycans bearing α2,3 terminal sialic acids (Sias) and absence of expression of α2,6-linked Sias in tumors of glial origin [27, 28]. Besides, high expression of structures containing terminal and core fucose has been described in GBM patients’ samples and also in a multistep model of glioma tumorigenesis [29]. In addition, increased expression of truncated O-GalNAc glycans has been identified in brain tumor tissues from patients with GBM compared to those from low-grade glioma and epilepsy patients [30]. In relation to N-glycosylation, normal glial cells have been described mainly presenting bi-antennary and oligomannose forms. In contrast, malignant glioma cells have shown more hybrid and complex structures [29, 31]. Moreover, a study with patients´ samples reported absence of high branched N-glycans with β1,6-GlcNAc in astrocytes from normal adult brain and high expression of them in GBM specimens [32].

The hypothesis of this work is that specific glycan patterns are associated with aggressive phenotypes of glioma. To address this question, we studied the phenotype of glycans and their *in vitro* biological role over a panel of low- and high-grade glioma cell lines, focusing on Lewis family, truncated O-GalNAc glycans, and oligomannose and complex high branched N-glycans. We demonstrated the association of high expression of terminal SLe^x^ with high-grade glioma, as part of complex N-glycans with β1,6-GlcNAc branching, and the potential involvement of histone acetylation in the resulting glycophenotype. Furthermore, this study stresses the role of sialylated and fucosylated complex N-glycans in the malignant behavior of high-grade glioma cells.

## RESULTS

**Figure 1:**
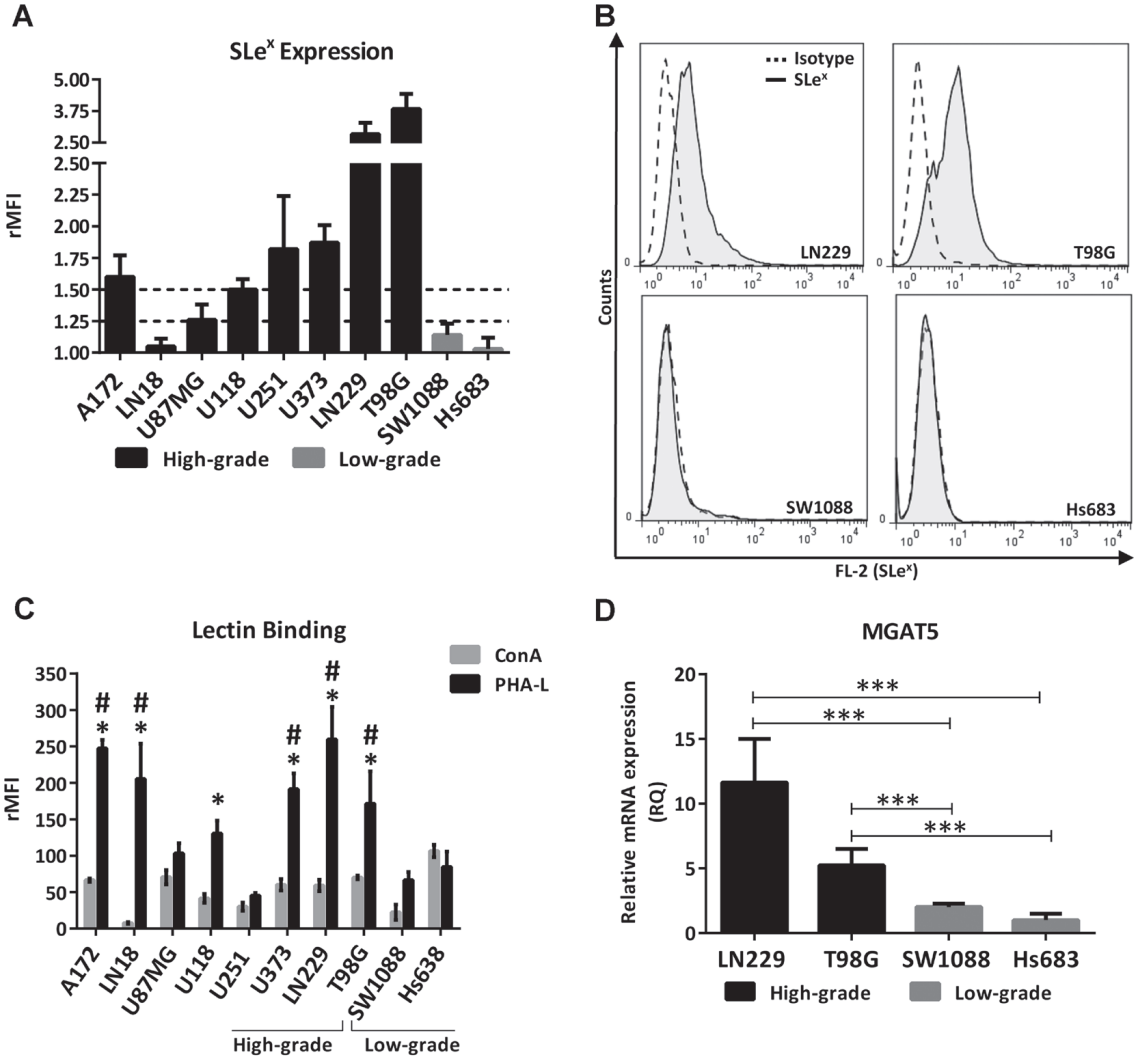
Glycophenotype characterization in high- and low-grade glioma cell lines. (**A**) SLe^x^ expression of high- and low-grade glioma cell lines evaluated by flow cytometry. Doted lines represent limits between low, medium and high expression. High expression was considered greater than 1.5 rMFI, medium between 1.25 and 1.5 rMFI, and low as lower than 1.25 rMFI. Data represent means ± S.D (rMFI) of three independent experiments. (**B**) Representative experiment for Sle^x^ expression. (**C**) Comparison of cell binding to lectins ConA and PHA-L by flow cytometry. ConA vs PHA-L: *p* < 0.05, *T* Test for A172, LN18, LN229, U118, U373 and T98G. PHA-L binding comparison against SW1088, ^*^ *p* < 0.05, *T* Test. PHA-L binding comparison against Hs683, ^#^ *p* < 0.05, *T* Test. (**D**) Relative comparison of MGAT5 transcripts levels between high- (LN229, T98G) and low-grade (SW1088, Hs683) glioma cell lines by qRT-PCR. Hs683 cell line was used as the reference sample (^***^ *p* < 0.001, ANOVA followed by Tukey’s multiple comparisons test).

As a first step to interrogate whether a differential profile of glycans is involved in the aggressiveness of glioma, we compared the expression of the Lewis glycan family (SLe^x^, Le^x^, SLe^a^, Le^a^, Le^y^ and Le^b^) and truncated O-GalNAc glycans (Tn, STn and T) between high- and low-grade human glioma cell lines by flow cytometry. Supplementary Table 1 presents means of fluorescence intensities relativized to the isotype control for each antibody (rMFI). In general, the high-grade cell lines showed medium (between 1.25 and 1.5 rMFI) or high expression (greater than 1.5 rMFI) of at least one Lewis glycan, in contrast to the low-grade lines that showed low expression (lower than 1.25 rMFI) of all the glycans analyzed. Le^a^ and Le^b^ were expressed in medium intensity by LN18 and U251. Le^y^ presented medium expression in A172 and T98G. SLe^a^ showed medium expression only in the LN229 cell line. In particular, SLe^x^ showed an association with the high-grade glioma cell lines. Figure 1A shows the expression of SLe^x^ in the panel of the glioma cell lines analyzed. High expression of SLe^x^ was found in A172, U118, U251, U373, T98G and LN229 cells, and medium expression was found in U87MG. Low expression of this terminal glycan was found in LN18 and in the two low-grade cell lines analyzed, SW1088 and Hs683. Figure 1B illustrates the expression of SLe^x^ of the two high-grade glioma cell lines with the highest expression and the two low-grade cell lines analyzed in this work. Regarding truncated O-GalNAc glycans, low expression was found in most of the cell lines evaluated. Only Tn antigen showed medium expression in the high-grade cell lines U87MG and T98G.

In line with the characterization of the glycan phenotype, we compared the N-glycan profile between high- and low-grade cell lines taking into consideration the binding to the lectins Concanavalin A (ConA) and Phytohemagglutinin-L (PHA-L) by flow cytometry (Figure 1C). ConA recognizes oligomannose-type N-glycans with high-affinity and complex-type bi-antennary N-glycans with low-affinity, while PHA-L recognizes structures with β1,6-GlcNAc branches of complex tri- and tetra-antennary N-glycans. Results showed that most of the glioma cell lines presented higher binding to PHA-L compared to ConA. Six out of eight high-grade glioma cell lines significantly overexpressed complex N-glycans bearing β1,6-GlcNAc branches compared with the high mannose type. In contrast, low-grade glioma cell lines did not show differences between ConA and PHA-L binding. Regarding the comparison between high- and low-grade cells, no significant differences in ConA binding were found between all glioma cell lines. Conversely, the majority of high-grade cell lines presented greater binding to PHA-L in comparison with the two low-grade cells analyzed in this study. Only U87MG and U251 did not present significant binding to PHA-L in comparison with the low-grade cell lines. In addition, we evaluated the transcription levels of MGAT5, a gene that encodes the GnT-V enzyme involved in the synthesis of PHA-L related structures. We compared the high-grade cell lines T98G and LN229, which showed both high expression of Sle^x^ and high binding to PHA-L, against the two low-grade cell lines SW1088 and Hs683, which presented low or null expression of Sle^x^ and lower binding to this lectin. As expected, Figure 1D shows the overexpression of MGAT5 in high-grade glioma in comparison with the low-grade glioma cell lines.

The N-glycan profile was also analyzed by High Performance Anion Exchange Chromatography with Fluorescence detection (HPAEC-FL) on four high-grade glioma cell lines. Supplementary Figure 1 shows a representative chromatogram quantification for LN229, U87MG, U251 and U373 in relation to the different species of glycans found in standards. The four cell lines presented a similar profile with high abundance of complex and sialylated N-glycans.

**Figure 2:**
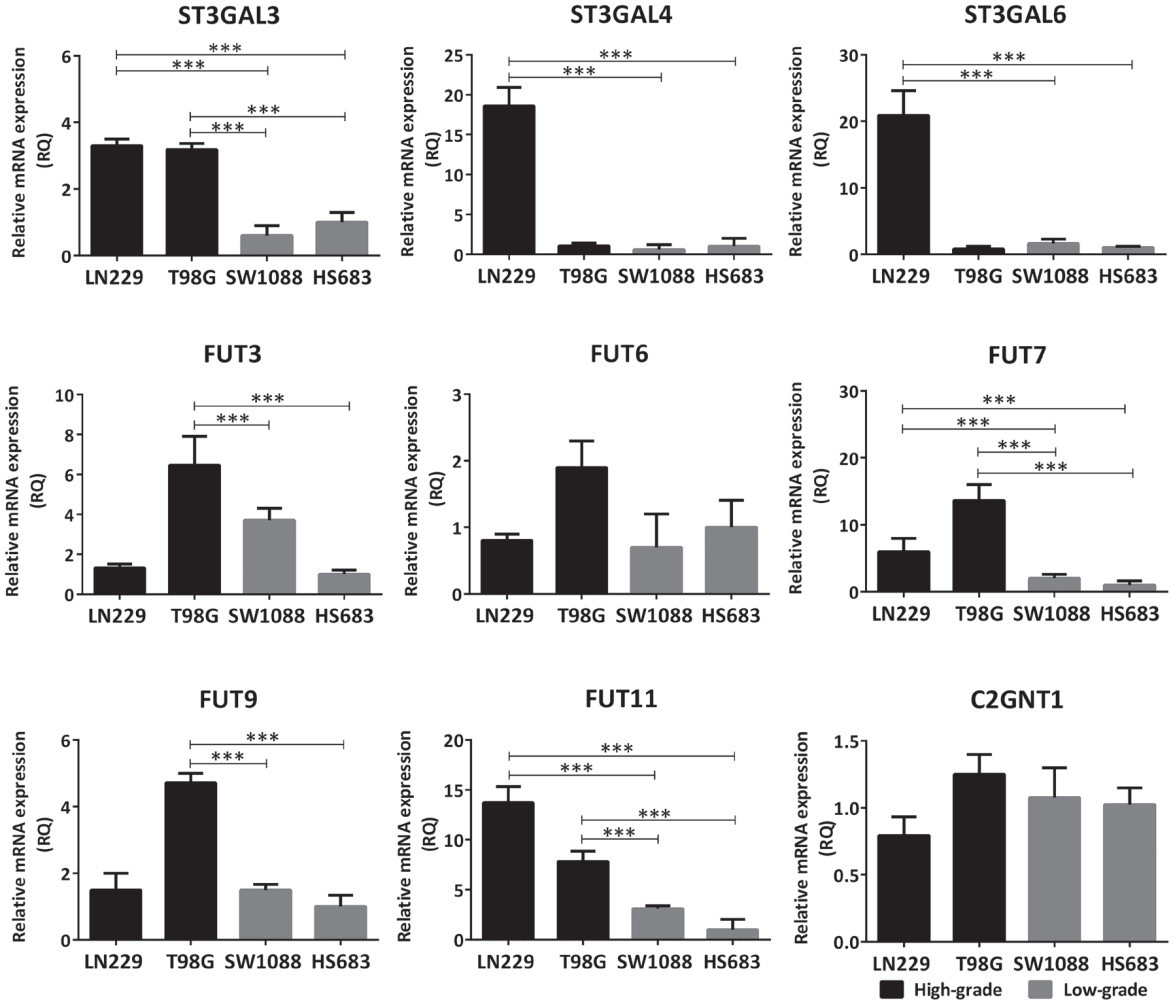
Comparison of transcription levels of glycosyltransferases involved in the biosynthesis of Lewis glycans between high- (LN229, T98G) and low-grade (SW1088, Hs683) glioma cell lines. The mRNA levels were analyzed by qRT-PCR. The relative amount of mRNA levels was normalized to the endogenous ACTB gene (human β-actin) expression and the cell line Hs683 was used as the reference sample. A significant biological result was considered at threefold difference between samples values. Data represent means ± S.D. of three independent experiments (^***^ *p* < 0.001, ANOVA followed by Tukey’s multiple comparisons test).

Expression of the glycosyltransferases involved in the biosynthesis of Lewis glycans was assessed by qRT-PCR, comparing high-grade (LN229, T98G) and low-grade (SW1088 and Hs683) cells in relation to their Sle^x^ expression. Lewis biosynthetic pathway is illustrated in Supplementary Figure 2. In general, the high-grade lines overexpressed transcript levels of several sialyl- and fucosyltransferases of the Lewis glycans biosynthesis pathway, compared to low-grade cells (Figure 2). Both LN229 and T98G overexpressed FUT7/11 and ST3GAL3 mRNA levels, compared with SW1088 and Hs683. Moreover, T98G showed significant overexpression of FUT3/9 and LN229 showed significant overexpression of ST3GAL4/6 mRNA levels. In contrast to the differences of transcripts expression observed for terminal sialyl- and fucosyltransferases, there was no significant difference in mRNA expression of C2GNT1 between low- and high-grade glioma cell lines (Figure 2).

**Figure 3:**
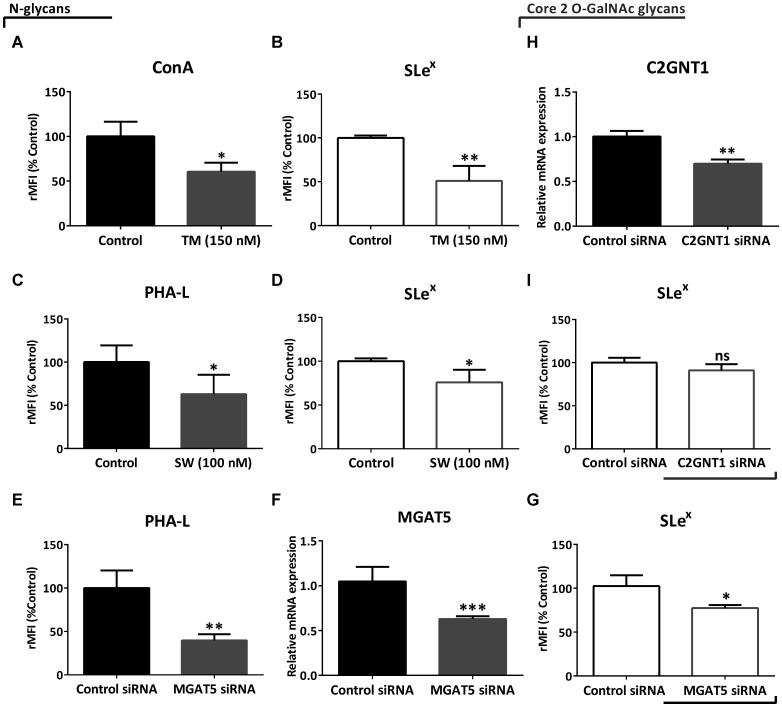
Evaluation of SLe^x^ expression under downmodulation of N- and core 2 O-GalNAc glycans in the high-grade glioma cell line LN229 by flow cytometry. (**A**) ConA binding and (**B**) expression of SLe^x^ after treatment with 150 nM TM for 24 h. (**C**) PHA-L binding and (**D**) expression of SLe^x^ after treatment with 100 nM SW for 24 h. (**E**) PHA-L binding, (**F**) MGAT5 transcripts levels and (**G**) expression of SLe^x^ after 48 h of MGAT5 or control siRNA transfection. (**H**) C2GNT1 transcripts levels and (**I**) expression of SLe^x^ after 48 h of C2GNT1 or control siRNA transfection. Data represent means ± S.D. of three independent experiments. (A, C, E, F, H and I) *T* Test. (B, D, G) Mann–Whitney. ^*^ *p* < 0.05, ^**^ *p* < 0.01, ^***^ *p* < 0.001.

In order to evaluate if Lewis glycans were part of N-glycans, we treated the high-grade glioma cell line LN229 with N-glycosylation inhibitors, Tunicamycin (TM) and Swainsonine (SW), and with a specific small interfering RNA (siRNA) targeting MGAT5. In particular, TM blocks the first step of N-glycan synthesis, while SW inhibits it at a later stage, mainly affecting the expression of complex N-glycans. For these and subsequent studies, the LN229 cell line was selected for being one of the models with the highest expression of Sle^x^. Overall, negative modulation of N-glycan synthesis produced a significant reduction in SLe^x^ levels in LN229 cells. Treatment with TM caused a decrease in the presence of N-glycans measured by ConA binding, and also a significant reduction in SLe^x^ expression by about 50% (Figure 3A and 3B). Since SW decreases the expression of complex N-glycans, we detected a reduction in PHA-L binding (Figure 3C). This treatment also reduced SLe^x^ expression by about 30% (Figure 3D). As regards controls, incubation with TM or SW caused a reduction in LN229 cell viability by 20% and 10%, respectively (Supplementary Figure 3). In addition, MGAT5 silencing provoked a significant decrease of PHA-L binding by about 60%, and it also decreased gene transcription level and SLe^x^ expression by about 30% (Figure 3E–3G). On the other hand, even though the silencing of C2GNT1 caused a significant decrease of mRNA levels, this treatment did not decrease expression of SLe^x^ in LN229 cells (Figure 3H and 3I).

**Figure 4:**
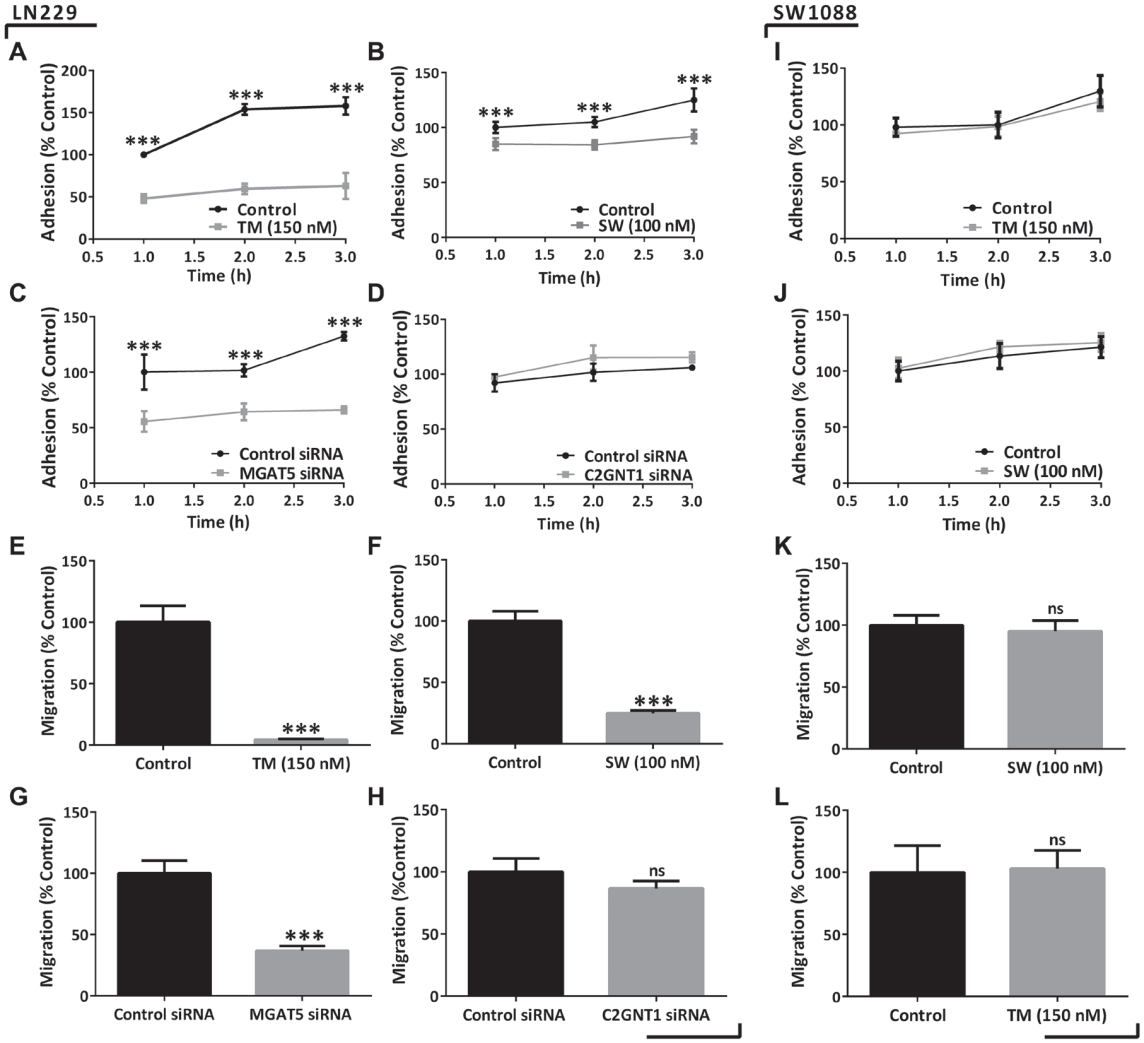
Evaluation of adhesion and migration capacities in high- and low-grade glioma cells under downmodulation of N- and core 2 O-GalNAc glycans. (**A**–**D**) LN229 cell adhesion measured after 24 h of treatment with 150 nM TM/100 nM SW or after 48 h of MGAT5/C2GNT1 silencing. (**E**–**H**) LN229 transwell cell migration in previously incubated with 150 nM TM/100 nM SW for 24 h or after 48 h of MGAT5/C2GNT1 silencing. (**I**–**L**) SW1088 adhesion and migration measured after treatment with 150 nM TM or 100 nM SW for 24 h. Data represent means ± S.D. of three independent experiments (ns *p* > 0.05, ^*^ *p* < 0.05, ^**^ *p* < 0.01, ^***^ *p* < 0.001, *T* Test).

Furthermore, the modulation of N-glycosylation impacted the *in vitro* cell behavior of the high-grade glioma cells LN229. Our results show that cell adhesion was significantly reduced by the treatments with TM or SW and also by the decreasing of MGAT5 mRNA levels (Figure 4A–4C). In addition, cell migration was inhibited after treatment with TM or SW, and MGAT5 silencing by 95%, 75%, and 60%, respectively (Figure 4E–4G). Conversely, C2GNT1 silencing did not affect LN229 adhesion and did not cause significant differences in the migration capacity of the LN229 cell line (Figure 4D and 4H).

Given the impact that N-glycans showed in high-grade-glioma we evaluated their participation on low-grade glioma cells. For these studies we selected SW1088 cell line, which showed low expression of SLe^x^. In contrast to the results obtained for high-grade, low-grade glioma cells did not show modulation in their malignant behavior after inhibition of N-glycosylation. The low-grade glioma cell line SW1088 was treated with the inhibitors TM or SW. No differences in adhesion and migration capacities were observed on this cell line as a result of these treatments (Figure 4I–4L). Incubation with TM or SW caused a decrease in cell viability by around 20%. Moreover, incubation with SW produced a reduction in the binding to PHA-L, observed by confocal microscopy (Supplementary Figure 4).

**Figure 5:**
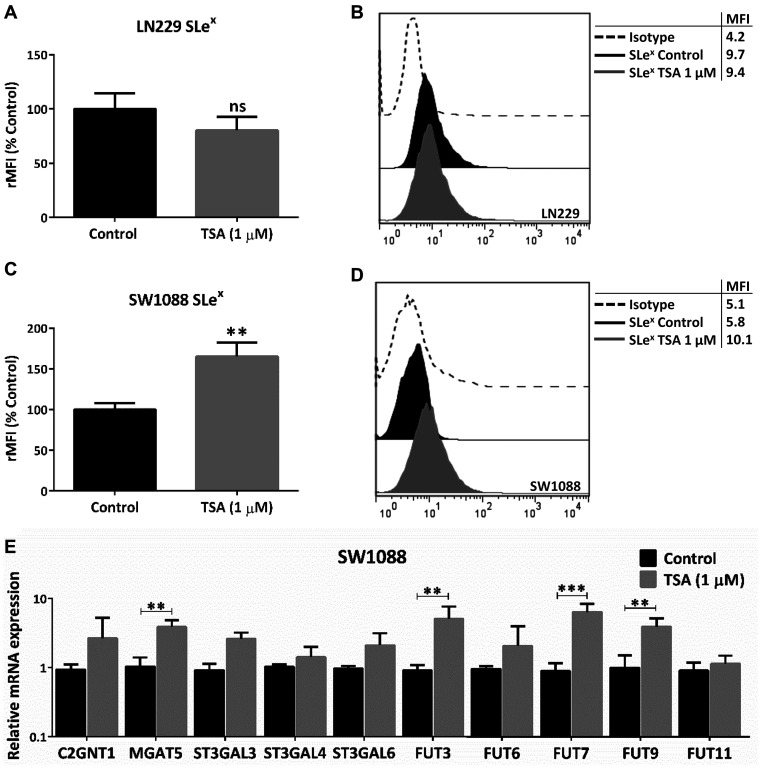
Evaluation of epigenetic regulation in glioma glycophenotype. LN229 and SW1088 cell lines were treated with 1 μM TSA for 24 h. Controls were incubated with DMSO. (**A**) SLe^x^ expression of LN229 cells (*p* > 0.05, *T* Test). (**B**) SLe^x^ expression of SW1088 cells (^*^ *p* < 0.05, *T* Test). (**C**) Relative comparison of glycosyltransferases mRNA levels between control and treated-SW1088 cells (^***^ *p* < 0.001, *T* Test). (**D**) Representative experiment of SLex expression of SW1088 cells after incubation with TSA or DMSO (**E**) Relative comparison of glycosyltransferases mRNA levels between control and treated-SW1088 cells (^***^ *p* < 0.001, *T* Test). Data represent means ± S.D. of three independent assays.

**Figure 6:**
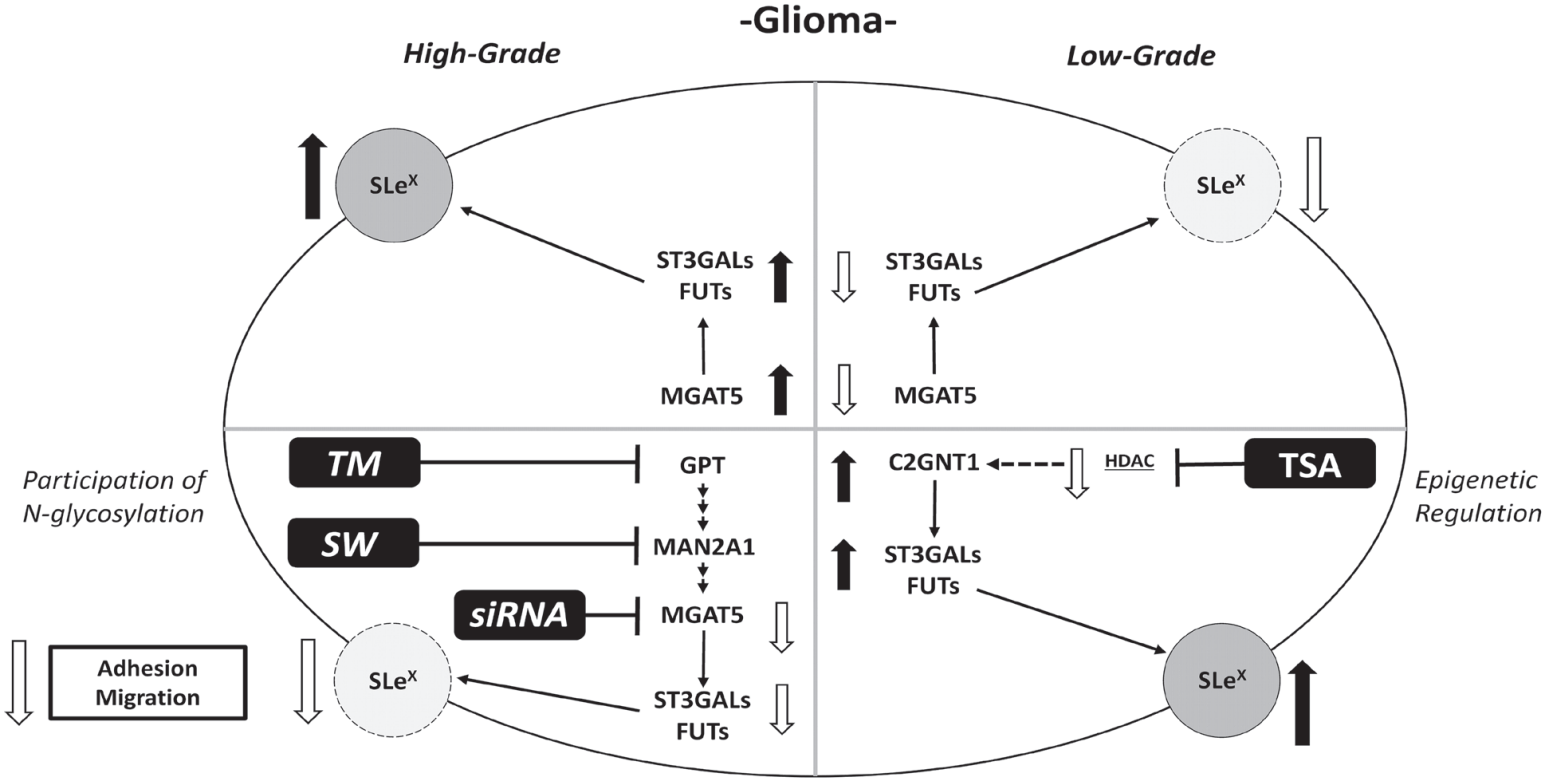
Schematic illustration of results obtained for Lewis glycans and expression of glycosyltransferases in treated and untreated high- and low-grade glioma cells.

Finally, we analyzed whether the described glycophenotype was regulated by epigenetic mechanisms involving histone acetylation. To address this, we treated LN229 and SW1088 cell lines with the histone deacetylases inhibitor Trichostatin A (TSA). No significant expression changes were observed in TSA-treated LN229 cells (Figure 5A and 5B). As a consequence of TSA incubation, we found a significant increase in the expression of SLe^x^ in SW1088 cells (Figure 5C and 5D). In addition, TSA treatment of this low-grade glioma cell line accounted for a significant increase in the transcription rate of MGAT5 and FUT3/7/9 (Figure 5E). Taking into consideration the results obtained in this study, we proposed a hypothetical model illustrated in Figure 6.

## DISCUSSION

Glioma is a disease for which targeted therapies could offer new therapeutic opportunities, especially for high-grade gliomas that inevitably recur within 2–3 cm from the original lesion after standard therapy [33]. Tumors tend to grow and infiltrate into the normal brain tissue, making the maximal resection and treatment an enormous challenge. For these reasons, the better understanding of glioma glycosylation, its participation in the cellular behavior, the main glycosyltransferases involved in the resulting phenotype and its regulation, may offer a new window to develop novel therapeutic approaches.

Our findings describe a differential glycophenotype between high- and low-grade glioma cells. These results were obtained by characterizing the expression of terminal and branching glycan patterns, interrogating the expression of genes involved in their biosynthesis, and evaluating their biological impact as well as their epigenetic regulation using a broad panel of human glioma cell lines.

Aggressive glioma cell lines present a complex N-glycan profile with sialylated forms. We found that high-grade glioma cells expressed more complex multi-antennary N-glycans in comparison with the oligomannose type. In relation to the complex type of N-glycans, we compared the expression of a well-documented cancer-associated structure, the β1,6-GlcNAc branching [34, 35]. In this work we found that this branching was significantly more expressed in high-grade compared with low-grade glioma. Besides, in accordance with the abundance of β1,6-GlcNAc branching, these cells overexpressed the related gene MGAT5 in comparison with low-grade glioma cell lines. Our findings are in line with previously reported results that show the overexpression of MGAT5 in a panel of glioblastoma cell lines and staining of β1,6-GlcNAc branching with the lectin PHA-L in glioblastoma specimens [32]. These results contribute with the hypothesis that β1,6-GlcNAc branching is a feature of the malignant transformation of glial tissue neoplasms.

In regard to the high abundance of terminal sialic acid structures, aggressive glioma cell lines showed high expression of terminal sialylated glycans. The characterization of cancer-associated glycans revealed that some members of Lewis group have shown to be highly expressed in high-grade glioma cells. In particular, SLe^x^ was highly detected in six out of eight high-grade glioma cell lines, in contrast to its absence observed in the low-grade glioma cells.

Interestingly, glioma cells have been described as devoid of α2,6 sialic acid and with high presence of this glycan in α2,3 linkage, which is, in fact, a building block for SLe^x^ along with terminal fucosylation. High-grade glioma cells showed overexpression of several sialyl- and fucosyltransferases involved in the biosynthesis of Lewis glycans and the high expression of SLe^x^ could be attributed to the expression of FUT7/11 and ST3GAL3. Similar results were recently reported by Park and colleagues, they found that the high-grade glioma cell line T98G expressed higher levels of α1,2 fucose and α2,3 sialic acid in comparison with the low-grade glioma cell line Hs683 [36]. In the same line, a microarray analysis of glyco-gene expression in human glioblastomas carried out by Kroes and colleagues showed the overexpression of the gene FUT3 within a panel of 11 glycosyltransferases overexpressed in human glioblastoma samples in comparison to normal brain tissue [37]. Additionally, Ashkani and colleagues reported the identification of glycosyltransferases gene expression profiles able to classify cancer types employing expression data taken from cancer patient samples in TCGA and, remarkably, ST3GAL3 gene was found to be highly overexpressed in GBM [38]. What is more, the grade IV glioma model developed by Furukawa and colleagues, exhibits an increase in the expression of N-glycans with core and terminal fucose residues in comparison with normal astrocytes [29]. This evidence supports the hypothesis that Lewis glycans are expressed in more aggressive glioma cells.

SLe^x^, terminal glycan associated with high-grade glioma, is mainly attached to complex N-glycans. Our results showed that blocking N-glycan biosynthesis by TM, SW or MGAT5 silencing caused a diminution of Sle^x^ in high-grade glioma cells. In contrast, since no significant changes in its expression were found under C2GNT1 silencing, this glycan seems to be absent from core 2 O-GalNAc structures.

Complex N-glycans were also found to modulate the *in vitro* behavior of high-grade glioma cells. The reduction of total N-glycans by treatment with TM, as well as the decrease of complex N-glycans by treatment with SW or silencing of MGAT5, resulted in a decrease in cellular adhesion and migration capacities. Similar results were described by Yamamoto et al. In this article, the authors demonstrated that N-glycans bearing β1,6-GlcNAc branching are associated with the invasion capacity of glioma cells [32]. Furthermore, Hassani et al. reported that the inhibition of GnT-V activity on orthotopic models of the high-grade SNB75 cell line resulted in a significant decrease in the proliferation and invasion capacities of tumor cells and also increased the overall survival of mice treated with standard temozolomide therapy [39].

In contrast to the results obtained for high-grade cells, the inhibition of N-glycan biosynthesis caused a minor impact in low-grade glioma cell behavior. The reduction of N-glycans in low-grade cell line SW1088 by treatment with TM or SW did not produce a decrease in both cell adhesion and migration. This suggests a lower participation of N-glycan structures in the aggressiveness of low-grade glioma. In this regard, the group of Rooprai described that compounds proposed as inhibitors of cell invasion, such as SW and flavonoids, did not modify the malignant phenotype of some low-grade glioma cell lines [40].

We also characterized the presence of other group of cancer associated glycans, the truncated O-GalNAc glycans. In this characterization we found medium expression of Tn in two high-grade glioma cell lines. In this line, the high-grade glioma model of Furukawa et. al showed a reduction of core 2 O-GalNAc glycans [29]. This group suggests that this inability to produce extended core 2 structures could result in the accumulation of truncated glycans. Accordingly, the comparison of the expression levels of C2GNT1, initiator of the core 2 of the O-GalNAc glycans, showed no difference between the high- and low-grade glioma lines. In the same light, silencing of C2GNT1 did not decrease SLe^x^ expression and did not modulate the *in vitro* cell adhesion and migration of high-grade glioma cells.

Regulation of gene expression by epigenetics involves several processes such as DNA methylation and post-translational modification of histones by methylation, acetylation, or phosphorylation, among others. Regarding the glycobiology of glioma, few reports associate epigenetic regulation with the expression of glycans and glycosyltransferases. One of the main examples is the ST6GAL1 expression loss caused by DNA methylation [41]. Regulation of chromatin acetylation levels is another mechanism associated with the regulation of gene expression in various types of tumors, including glioma. In this regard, HDACs have been associated with gene repression due to induction of a tight chromatin structure as consequence of the histone deacetylation [42]. As a preliminary, in this work, the induction of a scenario that promotes gene expression by using the broad-spectrum inhibitor of HDACs TSA reveled the epigenetic regulation of the glycan phenotype in glioma cells. The inhibition of HDACs in the low-grade SW1088 cell line induced an increase in the expression of SLe^x^ as well as it raised the expression of downregulated genes involved in β1,6-GlcNAc branching (MGAT5) and terminal fucosylation (FUT3/7/9). These results contribute to the limited understanding of epigenetic regulation in the phenotype of glioma cells, underscoring histone acetylation as a factor that needs further investigation.

The study of tumor glycosylation converges in the opportunity of developing novel targeted therapies. For example, the characterization of gliomas glycophenotypes could give insights into the tumor capacity to have an attractor or no non-attractor phenotype regarding infiltrative blood-borne cells. Among the multiple roles of glycosylation in cell biology, it is a main determinant in the extravasation of blood-borne cells into GBM tissue, such as bone marrow-derived human mesenchymal stem cells (BM-hMSCs). In this regard, Wildburger and colleagues provided evidence for differential glycomic profiles in pre-clinical glioma stem cell xenograft (GSCX) models with the attractor and non-attractor phenotype. In the aforementioned study, both glycotranscriptomic analysis and nLC-ESI-MS data showed significant heterogeneity within the attractor phenotype and the enrichment of high mannose type N-glycan biosynthesis in the non-attractor phenotype. Additionally, the authors reported prevalence of terminal sialic acid-containing N-glycans in non-attractors and terminal galactose and N-acetyl-glucosamine N-glycans in attractors. These results contribute to the understanding of cell-mediated therapeutic delivery for the advancement of GBM treatment [43].

Our findings demonstrate a differential pattern of terminal Lewis glycans in high-grade glioma cell lines, and their association with the overexpression of different glycosyltransferases involved in their biosynthesis. These glycans would be part of complex N-glycan structures, which have a relevant role in key events of the glioma tumor biology such as cell adhesion and migration. Furthermore, there are epigenetic regulatory mechanisms that potentially mediate the expression of this glycophenotype. Since data from cell lines may be interpreted provisionally, evaluation of the glycosylation profile as well as glycosyltransferases expression should be carried out on primary glioblastoma tissue, the gold standard for relevance to future diagnostics or therapeutics.

Profiling glycosylation changes on the cell surface of tumoral cells can lay the foundations for the discovery of novel markers as well as therapeutic targets by defining specific glycan structures associated to a biological impact. This work emphasizes the role of terminally sialylated and fucosylated complex N-glycans in the malignant behavior of high-grade glioma cells and opens new windows to further investigate the potential candidates behind the characterized glycophenotype.

## MATERIALS AND METHODS

### Cell lines

This study was carried out using a panel of high- and low-grade human glioma cell lines. The former group include the grade IV (GBM) cell lines LN229, LN18, U87MG, U251, U373, T98G, U118MG and A172 [44–49]. The low-grade group includes SW1088 and Hs683 cell lines, which derived from a diffuse astrocytoma and an oligodendroglioma, respectively [50, 51]. The T98G and LN229 cell lines were purchased from the American Type Culture Collection (ATCC, USA). LN18, U118, A172, SW1088 and Hs683 were kindly provided by GlaxoSmithKline, USA. U87MG, U251 and U373 were kindly provided by Dra. Marianela Candolfi, Institute of Biomedical Research, University of Buenos Aires, Argentina (INBIOMED, UBA-CONICET). LN229, LN18, U87MG, U251, U373, T98G and U118 were grown in Dulbecco’s Modified Eagle’s Medium (DMEM) (Gibco, USA), while SW1088, Hs683 and A172 were grown in Roswell Park Memorial Institute (RPMI) 1640 Medium (Sigma, USA). All mediums were supplemented with 80 μg/ml of gentamicin (Northia, Argentina) and monolayers were routinely sub-cultured with Trypsin-EDTA solution (Gibco, USA), following standard procedures. Cell lines were supplemented with 10% fetal bovine serum (FBS) (Gibco, USA), with the exception of LN18, which was supplemented with 5% FBS. Cell cultures were maintained at 37°C in a humidified atmosphere of 5% CO2 and tested for Mycoplasma every three months by DAPI staining (Vector, USA). Cells passages lower than 20 were used for the described experiments.

### Flow cytometry assay

Cell lines were cultured without FBS for 24 h, collected by enzyme-free cell dissociation buffer (Thermo Fisher Scientific, USA) and incubated with glycan-specific monoclonal antibodies (Abs) or isotype control on ice for 30 min. The mAbs used were Le^x^ (4E10, Novus Biologicals, USA), SLe^x^ (CSLEX1, BD Pharmingen, USA), Le^a^ (Abcam, UK), SLe^a^ (KM231, Millipore, USA), Le^b^ (2-25LE, Abcam, UK), Le^y^ (F3, Abcam, United Kingdom), T (Thomsen-Friedenreich Antigen, SPM320, Novus Biologicals, USA), Tn (Tn 218, Abcam, UK), STn (B72.3, Santa Cruz, USA), Mouse IgG1 Isotype Control (Thermo Fisher Scientific, USA), Mouse IgM Isotype Control (Dako, USA). Cells were washed with phosphate buffered saline (PBS) and incubated with Polyclonal Goat Anti-Mouse Phycoerythrin (PE) labeled anti-mouse immunoglobulins (Dako, USA) on ice for 30 min. For lectins binding, biotinylated ConA or PHA-L (Vector Laboratories, USA) were incubated with fluorescein-streptavidin (Vector Laboratories, USA) in 1% Bovine Serum-Albumin (BSA) (Sigma, USA) in PBS on ice for 30 min. Then, cells were incubated with the lectin-streptavidin complex on ice for 30 min. For mAbs or lectins, acquisition was carried out by a FACSCalibur flow cytometer (Becton Dickinson, USA) and analyzed by the FlowJo^®^ software. Data was analyzed as median fluorescence intensity relativized to isotype control (rMFI). Expression was defined as: high when it was over 1.5 rMFI; as medium if between 1.25 and 1.5 rMFI; and as low if lower than 1.25 rMFI.

### Quantitative RT-PCR (qRT-PCR)

After 24 h of serum starvation, total RNA from 1 × 10^6^ cells was purified with the EasyPure^®^ RNA Kit (TransGen Biotech, China) according to the manufacturer’s protocol. RNA was reverse transcribed with Super Script™ III (Thermo Fisher Scientific, USA) following the manufacturer’s protocol. Primers were designed using the Primer3 plus software (http://www.bioinformatics.nl/cgi-bin/primer3plus/primer3plus.cgi) and The Basic Local Alignment Search Tool (BLAST). Primers were manufactured by Invitrogen (USA) and their sequences are provided in Supplementary Table 2. The qRT-PCR experiments were carried out with Power SYBRTM Green PCR Master Mix (Thermo Fisher Scientific, USA) and StepOne Real-Time PCR System (Applied Biosystems, USA). The thermal cycling conditions used were the following: 48°C for 30 min, 95°C for 10 min, 40 cycles of 95°C for 15 seconds followed by 60°C for 60 seconds. Each sample was analyzed in triplicate and mean cycle threshold values (Ct) were used for further analysis. ACTB gene (Human β-actin) mRNA levels were used for Ct normalization. Relative quantification (RQ) values were calculated as 2^−ΔΔCt^. A significant biological result was assumed when a threefold difference between samples values was obtained.

### Gene silencing

The expression of the genes C2GNT1 and MGAT5 was downmodulated by small interfering RNA (siRNA). C2GNT1, MGAT5 and a scramble siRNAs (control) were obtained from Origene (USA). Gene-targeting constructs are composed by 3 unique 27mer siRNA duplexes (Locus ID 2650 for C2GNT1 and ID 4249 for MGAT5), and control siRNA is a Trilencer-27 Universal Scrambled Negative Control. Cells were plated on 60 mm dishes, grown until approximately 80% of confluence and then transfected with C2GNT1, MGAT5 or control siRNA using Lipofectamine 2000 (Thermo Fisher Scientific, USA) following the manufacturer’s instructions. For adhesion and migration assays, cells were collected and plated 24 h after transfection and for qRT-PCR, cells were processed 48 h after transfection.

### Inhibition of N-glycosylation

Cells were cultured in their proper medium with 150 nM Tunicamycin (TM) (Merck KGaA, Germany) or 100 nM Swainsonine (SW) (Sigma, USA) for 24 h. Controls were incubated with an equivalent volume of DMSO or PBS, respectively [52, 53].

### Trichostatine A treatment

Trichostatine A (TSA) was provided by Dr. Norberto W. Zwirner, Laboratory of Pathophysiology of Innate Immunity, Institute of Experimental Biology and Medicine (IBYME), Argentina. Cells were incubated with 1 μM TSA for 24 h, controls were incubated with an equivalent volume of DMSO [54].

### Cell adhesion assay

Cell adhesion was measured by a colorimetric method using crystal violet staining in LN229 or SW1088 cell lines. After 48 h of siRNA transfection (MGAT5, C2GNT1 or control) or 24 h of incubation with TM or SW, cells were harvested with an enzyme-free cell dissociation buffer and seeded at a concentration of 4 × 10^4^ cells/well in complete medium in a 96-well plate. After 1–3 h of incubation at 37°C, cells were washed with PBS, and non-adherent cells were removed by aspiration. Adherent cells were stained with a 0.5% (w/v) crystal violet solution with 20% (v/v) methanol. After washes, the dye was solubilized by adding the solution containing 10% (v/v) methanol and 5% (v/v) acetic acid, and the absorbance was measured at 595 nm by a microplate reader.

### Cell proliferation assay

Cells were plated in 96-well flat bottom plates at a density of 5 × 10^3^ cells/well in 200 μl of corresponding medium supplemented with 10% FBS. The proliferative effect of TM and SW was evaluated after 24 h. Cell proliferation was measured by crystal violet assay as previously described.

### Cell migration assay

After overnight starvation, 1 × 10^5^ LN229 or SW1088 cells previously transfected with siRNA (MAGAT5, C2GNT1 or control) for 48 h, or treated with TM or SW for 24 h, were seeded into the Transwell^®^ inserts with 8 μm pore (Corning, USA) in serum-free medium. The lower chamber was filled with 10% FBS containing medium. Stationary cells were removed from the upper surface of the membranes with a cotton swab. Cells that migrated to the lower surface were fixed and stained with crystal violet. Migrating cells were counted in five randomly selected fields and normalized to control.

### HPAEC-FL

LN229, U87MG, U251 or U373 cells were lysed with RIPA buffer (1M TrisHCl, 5M NaCl, 0.1% (v/v) Triton-X 100). Total protein quantification was carried out by colorimetric method with bicinchoninic acid using the Pierce™ BCA Protein Assay Kit (Thermo Fisher Scientific, USA) according to the manufacturer’s instructions. N-glycans from 50 μg of total protein lysate were released using the recombinant PNGase F kit (New England Biolabs, USA). Samples were reacted with 2-Aminobenzamide (2-AB) dissolved in a cyanoborohydride reaction mixture. 2-AB-labeled glycans were separated by HPAEC and detected by fluorescence using sodium hydroxide and sodium acetate gradient. The N-glycans from RNAse b and Fetuin A were used as standards. Chromatograms were analyzed using Chromeleon™ 6.8 Chromatography Data System software (ThermoFisher Scientific, USA).

### Immunofluorescence

5 × 10^5^ SW1088 cells were seeded on coverslips (Thermo Fisher Scientific, USA) in 6-well plates. Cells were washed with PBS and fixed for 10 min with 4% formalin in PBS. After a wash with PBS, cells were incubated with 1% BSA in PBS for one hour. Then cells were incubated with 0.5 μg of biotinylated ConA at 4°C overnight. Following tree washes with PBS, cells were incubated with 0.5 μg of fluorescein-streptavidin (Vector Laboratories, USA) in 50 μl of PBS for 1 h at room temperature. Finally, the coverslips were washed three times with PBS and mounted with DAPI (Vector Laboratories, USA). The images were captured by inverted Confocal Spectral Scanning Microscope TCS SP8 (Leica, Germany).

### Statistical analysis

Statistical significance was calculated using Prism 6 statistical software (GraphPad, Inc. California, USA). The data presented in this study is expressed as mean values ± SD. Normality test was performed prior to statistical test. For comparisons between two independent samples, *T*-Test or Mann–Whitney was used. For multiple comparisons between experimental groups ANOVA (followed by Tukey’s multiple comparisons test) was performed. The data correspond to at least three independent experiments. A statistically significant value was defined as *p* < 0.05.

## SUPPLEMENTARY MATERIALS



## Abbreviations

C2GNT1: Core 2 Beta1,6 N-Acetylglucosaminyl transferase
FUT: fucosyltransferase
FBS: Fetal Bovine Serum
GalNAc: N-Acetylgalactosamine
GlcNAc: N-Acetylglucosamine
GBM: glioblastoma
GnT-V: N-acetylglucosaminyltransferase V
HDACs: histone deacetylases
HPAEC-FL: High Performance Anion Exchange Chromatography with Fluorescence detection
Le^a^: Lewis A
Le^b^: Lewis B
Le^x^: Lewis X
Le^y^: Lewis Y
PBS: phosphate buffered saline
PE: Phycoerythrin
qRT-PCR: Quantitative RT-PCR
rMFI: relative Median Florescence Intensity
RQ: Relative quantification
Sia: sialic acid
siRNA: Small interfering RNA
SLe^a^: Sialyl Lewis A
SLe^x^: Sialyl Lewis X
ST: sialyltransferase
STn: SialylTn
SW: Swainsonine
TM: Tunicamycin
TSA: Trichostatin A
T: Thomsen–Friedenreich Antigen
WHO: World Health Organization
