# Supplementary material for: Terminally sialylated and fucosylated complex N-glycans are involved in the malignant behavior of high-grade glioma
Source: Oncotarget. 2020 Dec 29;11(52):4822–35. doi: 10.18632/oncotarget.27850 (PMC7779250; doi:10.18632/oncotarget.27850)
Supplement: Supplementary file 1 [file oncotarget-11-4822-s001.pdf]

Terminally sialylated and fucosylated complex N-glycans are involved in the malignant behavior of high-grade glioma

SUPPLEMENTARY MATERIALS

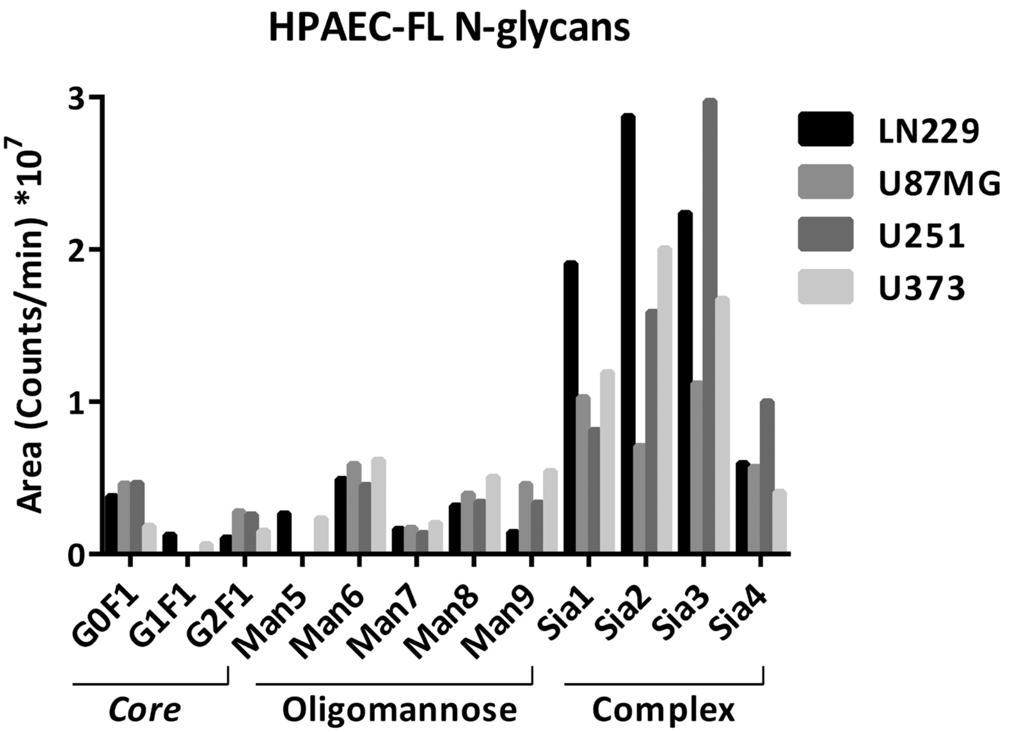

Supplementary Figure 1: N-glycans analysis by HPAEC-FL of LN229, U87MG, U251 and U373 cell lines. G: Galactose; F: Fucose; Sia: sialic acid.

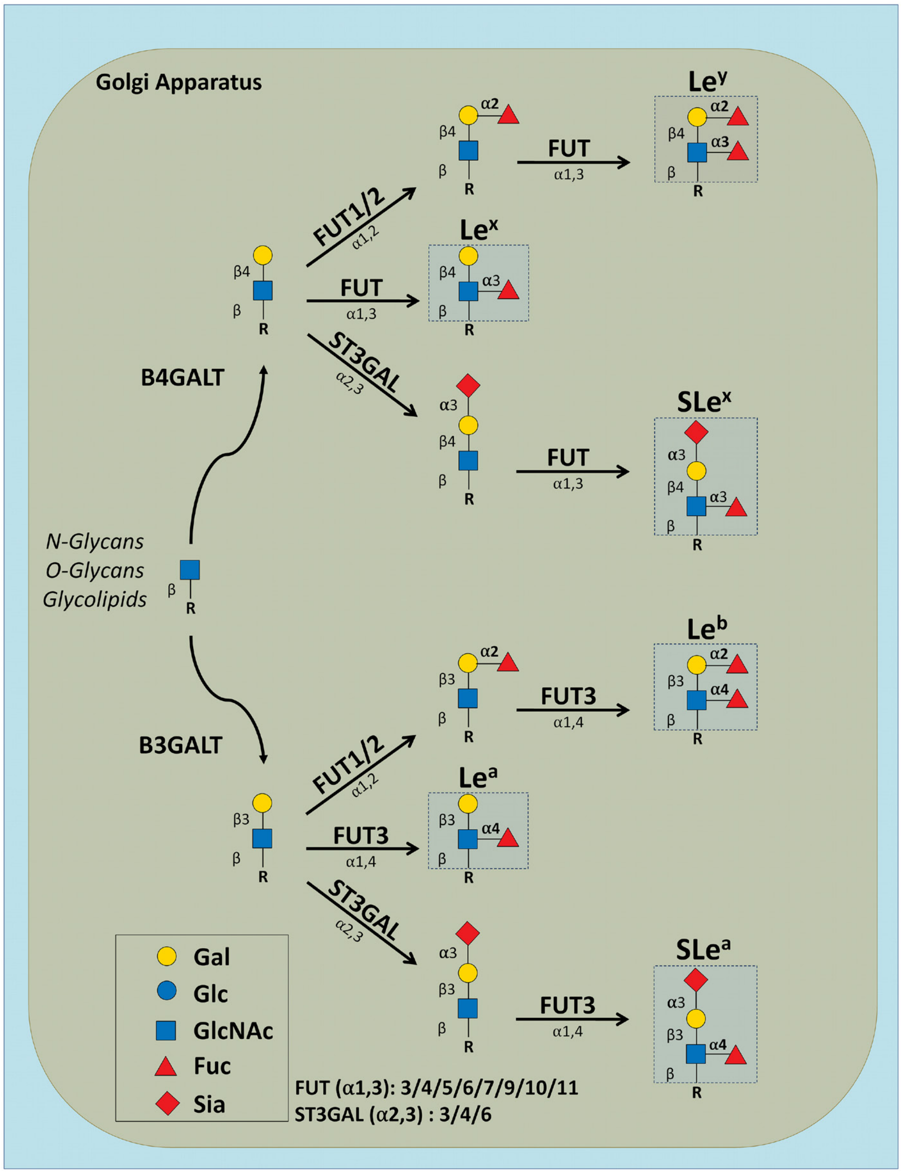

Supplementary Figure 2: Biosynthetic pathway of Lewis glycans family.

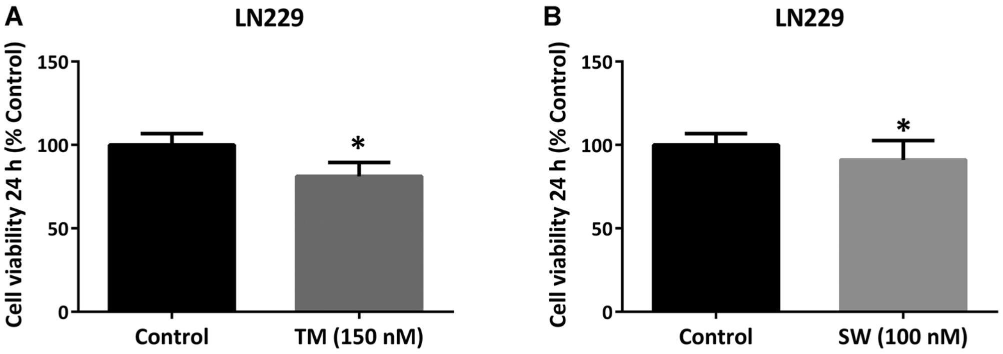

Supplementary Figure 3: Evaluation of LN229 cell viability after 24 h of inhibition of N-glycosylation. (**A**) Treatment with 150 nM of TNM. (**B**) Treatment with 100 nM of SW. Data represent rMFI means ± S.D. of triplicate determinants ($^{*}p < 0.05$, *T* Test).

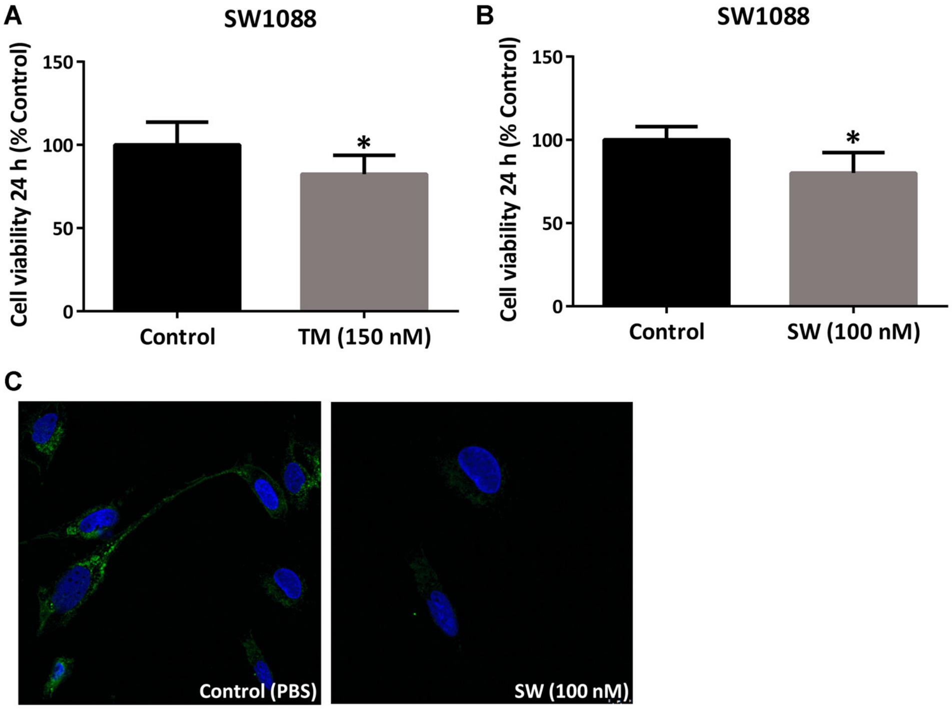

Supplementary Figure 4: Inhibition of N-glycosylation of low-grade SW1088 glioma cell line. Cell viability after treatment with 150 nM of TNM (**A**) or 100 nM of SW (**B**) for 24 h. Data represent rMFI means ± S.D. of triplicate determinants ($^{*}p < 0.05$, *T* Test.). (**C**) Confocal microscopy of untreated SW1088 cells (PSB control) and treated (100 nM SW) cells, stained with PHA-L-fluorescein.

Supplementary Table 1: Glycan expression evaluated by flow cytometry in the high- and low-grade glioma cell lines

	High-Grade Glioma								Low-Grade Glioma	
Lewis Glycans	A172	LN18	LN229	U87MG	U118	U251	U373	T98G	SW1088	HS683
Le^a	0.97 ± 0.12	*1.26 ± 0.09*	0.99 ± 0.20	1.07 ± 0.02	0.99 ± 0.04	*1.33 ± 0.05*	1.01 ± 0.05	0.99 ± 0.10	1.02 ± 0.05	1.01 ± 0.12
Sle^a	0.90 ± 0.12	0.94 ± 0.20	*1.26 ± 0.09*	1.02 ± 0.03	1.01 ± 0.03	1.17 ± 0.06	1.03 ± 0.05	1.01 ± 0.10	1.11 ± 0.06	1.04 ± 0.10
Le^b	0.96 ± 0.10	*1.25 ± 0.11*	1.19 ± 0.06	1.16 ± 0.12	1.02 ± 0.10	*1.32 ± 0.12*	1.01 ± 0.10	1.01 ± 0.08	1.15 ± 0.06	1.12 ± 0.10
Le^x	0.95 ± 0.09	1.04 ± 0.10	1.12 ± 0.11	0.97 ± 0.09	1.05 ± 0.04	1.21 ± 0.04	1.02 ± 0.12	1.05 ± 0.06	0.97 ± 0.02	1.16 ± 0.05
SLe^x	**1.60 ± 0.17**	1.05 ± 0.06	**2.84 ± 0.45**	*1.26 ± 0.12*	**1.50 ± 0.08**	**1.82 ± 0.42**	**1.87 ± 0.14**	**3.83 ± 0.60**	1.14 ± 0.09	1.03 ± 0.09
Le^y	*1.47 ± 0.20*	1.15 ± 0.06	1.18 ± 0.07	1.10 ± 0.13	1.16 ± 0.01	1.21 ± 0.06	1.02 ± 0.03	*1.48 ± 0.12*	1.04 ± 0.10	1.09 ± 0.07
**Truncated O-Glycans**										
T	1.14 ± 0.10	1.01 ± 0.05	1.05 ± 0.06	1.09 ± 0.10	0.95 ± 0.03	0.94 ± 0.09	1.12 ± 0.08	1.05 ± 0.10	1.01 ± 0.03	ND
Tn	1.07 ± 0.12	0.99 ± 0.10	0.99 ± 0.10	*1.32 ± 0.09*	0.97 ± 0.10	1.01 ± 0.08	1.23 ± 0.02	*1.29 ± 0.08*	1.02 ± 0.10	ND
STn	1.04 ± 0.09	1.05 ± 0.10	1.06 ± 0.10	0.99 ± 0.06	1.02 ± 0.10	1.24 ± 0.20	0.96 ± 0.10	1.18 ± 0.06	1.14 ± 0.10	ND

The data represent rMFI means ± S.D of three independent experiments. ND: Not Determined. High expression was considered greater than 1.5 rMFI, medium between 1.25 and 1.5 rMFI, and low as lower than 1.25 rMFI.

Supplementary Table 2: Sequence of the primers used for the analysis of glycosyltransferases by quantitative RT-PCR

Gene	Forward Primer	Reverse Primer
ACTB	CAAGATCATTGCTCCTCCTG	AGCACTTGCGGTGCACGATG
C2GNT1	AAGCAGTTGCCAGGTTTG	ACACTGAGCGCACATGGAC
FUT3	GGGTTAAGAGCTCAGAGTTCAGAC	AGCAGCAATTTCCCTCAACCC
FUT4	TTGCACAGCTAGCAATTGGG	ATTCAGGAAACCGCCTCAAC
FUT5	AAGCCACATCGCATTGAAGC	TGGAGCCCGGACATCCTTTG
FUT6	CACCTCCGAGGCATCTTCAACTG	CGTTGGTATCGGCTCTCATTCATG
FUT7	CACCTCCGAGGCATCTTCAACTG	CGTTGGTATCGGCTCTCATTCATG
FUT8	GGTCGAGCTTCCCATTGTAG	GCGAGGTCTTCTGGTACAGC
FUT9	CTTACCGCCGTGATTCAGAT	AATGCTTGCCCGTAGGTATG
FUT11	CTCTTGGCTTTCTTGTCC	ATGACGGAGTGATTGTTC
ST3GAL3	TCTCCGCTGTGGTCATTTAGG	AGTACCAGAAAGAGGCAGAGG
ST3GAL4	AGTAGAAAACAACCCAGACAC	AGAGGTTGAGAATCCGAA
ST3GAL6	AGAGTCCTTTGCACTACTATGG	CACTGTTAGCATCATCTTCTGAG
MGAT5	AGCCTGAAAGCAGCTCCAT	GCCAGTGCCTTGATGTACCT

Human ACTB gene (β-actin) was used as endogenous control.
